# Data-driven decarbonisation pathways for reducing life cycle GHG emissions from food waste in the hospitality and food service sectors

**DOI:** 10.1038/s41598-022-27053-6

**Published:** 2023-01-09

**Authors:** I Kit Cheng, Kin K. Leong

**Affiliations:** 1 Department of Physics and Astronomy, University College London, London, UK; 2 Institute of Environmental Management and Assessment (IEMA), March, Cambridgeshire, UK

**Keywords:** Environmental social sciences, Sustainability

## Abstract

The Hospitality and Food Service (HaFS) sectors are notoriously known for their contribution to the food waste problem. Hence, there is an urgent need to devise strategies to reduce food waste in the HaFS sectors and to decarbonise their operation to help fight hunger, achieve food security, improve nutrition and mitigate climate change. This study proposes three streams to decarbonise the staff cafeteria operation in an integrated resort in Macau. These include upstream optimisation to reduce unserved food waste, midstream education to raise awareness amongst staff about the impact of food choices on the climate and health, and finally downstream recognition to reduce edible plate waste using a state-of-the-art computer vision system. Technology can be an effective medium to facilitate desired behavioural change through nudging, much like how speed cameras can cause people to slow down and help save lives. The holistic and data-driven approach taken revealed great potential for organisations or institutions that offer catering services to reduce their food waste and associated carbon footprint whilst educating individuals about the intricate link between food, climate and well-being.

## Introduction

Food loss and waste (FLW) is a significant global problem with estimates by the FAO^1^ suggesting that one-third of edible food produced for human consumption is wasted globally each year. In the context of national emissions, if FLW were a country, it would be the third largest greenhouse gas (GHG) emitter in the world after China and US^2^. Reducing global FLW would significantly reduce GHGs in the atmosphere, playing a major role in tackling climate change. The issue becomes even more critical when the world population is projected to reach 9.8 billion by 2050^3^, while a staggering 11.3% people still go hungry on a daily basis^4^. This puts an ever-increasing demand on the global food system to feed a growing population.

Natural resources such as fresh water and fertile soil needed for food production may be depleted faster than it could be replenished. Their preservation is critical for global food security. Hence, zero hunger, clean water and sanitation, responsible consumption and production are part of the 17 Sustainable Development Goals (SDGs) formulated in 2015 and adopted by many developed and developing countries^5^. The SDG target 12.3 is set to ‘halve per capita global food waste at the retail and consumer levels and reduce food losses along production and supply chains, including post-harvest losses by 2030’. The International Food Waste Coalition (IFWC) reported the latest average food waste in the Hospitality and Food Service (HaFS) sector of 115 g per dining cover in 2021, noting that this value was derived from an over-representation of sites in France^6^. In April 2021, China passed a food waste law for preventing food waste, safeguarding national food security, conserving resources, protecting the environment, and promoting sustainable economic and social development^7^.

### Food waste in HaFS sectors and its associated impacts

By definition, food loss and waste (FLW) refer to the decrease in mass or nutritional value of edible food parts throughout the supply chain that was intended for human consumption^8^. According to the FAO^9^, food losses occur from post-harvest up to, but not including, the retail stage. It is often unintentional and largely due to inadequate infrastructure and transportation to maintain the food quality from farm to retail. On the other hand, food waste occurs at the retail and consumer stages of which the HaFS sectors contribute significantly. It is often due to high food standard requirements, expired food from over supply or under demand and wasteful consumer behaviour. The HaFS sectors have a major role to play in the reduction of food waste, especially organisations or educational institutions with in-house cafeteria patronised by thousands of individuals every day.

**Figure 1:**
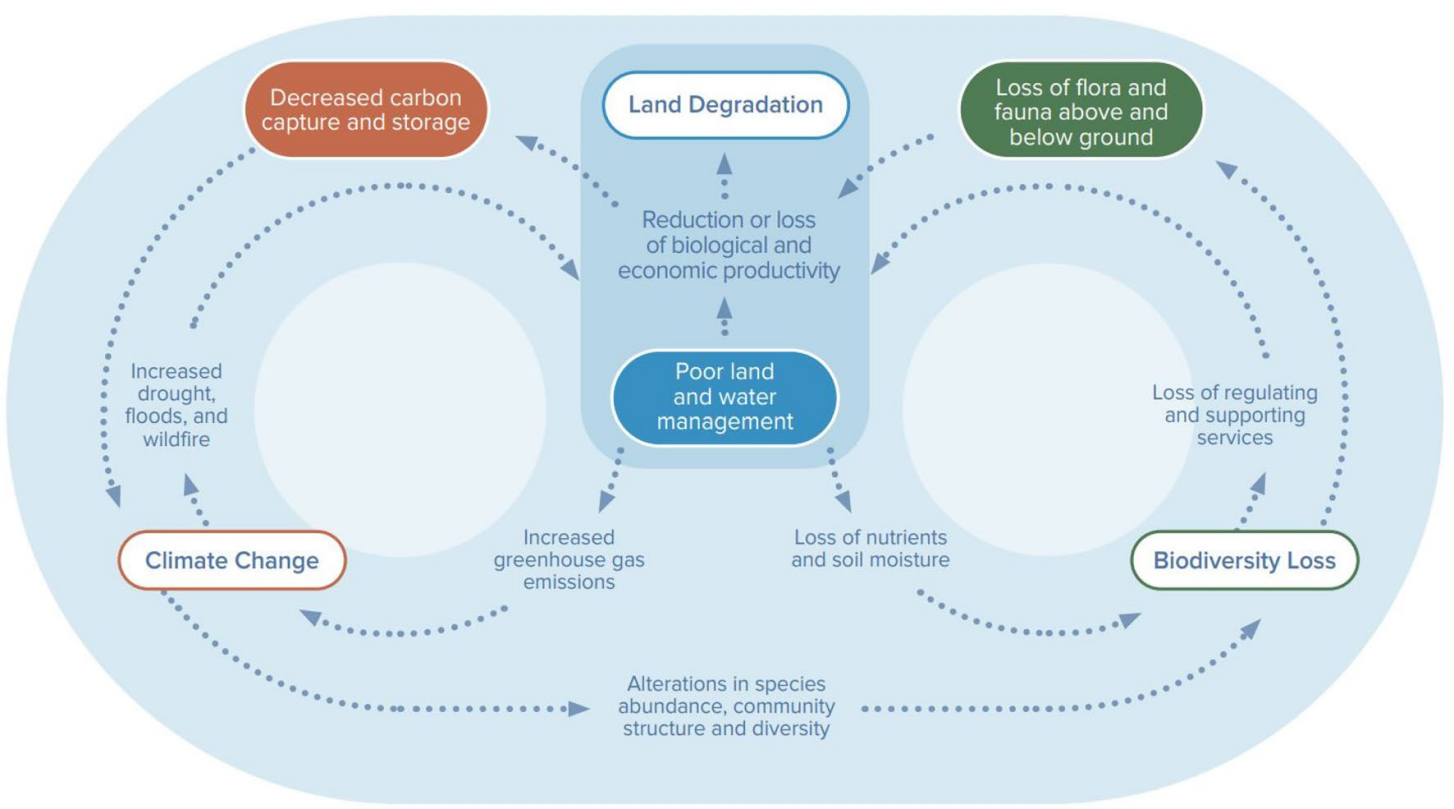
Feedback loops between land degradation, climate change, and biodiversity loss. Taken from Figure 1.3 in The Global Land Outlook UNCCD (2022)^19^.

Food waste is not just about the waste itself, but also the negative environmental impacts of the carbon and water footprint^10^, the loss of biodiversity^11^, and the drain on the economy^12^. Figure 1 illustrates how the positive feedback loop of poor land and water management accelerates climate change, biodiversity loss and land degradation. The problem of food waste is inextricably linked to climate change through the release of GHGs. The challenges posed by climate change include water and food security^13^, as well as extreme weather such as increased frequency and intensity of climate-related disasters which threaten public health^14^ and cause massive economic loss^15^. For Macau, intense floods and typhoons are particularly dire as seen from the devastating impacts of Typhoon Hato in 2017^16^. Sources of GHG emissions in our food system include deforestation for agriculture, fertilisers for plants, rice farming, livestock grazing, livestock manure and fossil fuels used in food production and supply chains^17^. Global food production is estimated to account for 26% of global GHG emissions. Yet, one-quarter of that comes from FLW, that is food lost in the supply chains and consumer waste, not including food losses on the farm during production and harvesting^18^. A significant reduction of GHG emissions could be achieved by optimising the food supply chain which could ultimately help limit the global temperature rise to below 2 $^\circ$C as set out by the Paris Agreement^17^.

The food waste data from the integrated resort in this study shows two main patterns. Firstly, ‘unserved food’ accounts for the majority of back kitchen food waste, which also includes waste from trimmings, spoilage, damage, and cooking error. This implies that the chefs are overestimating the demand in the time frame the food is being served. This problem could be tackled by optimising supply based on demand. Secondly, the ‘plate waste’ category accounted for the majority (over 90%) of total food waste in the staff cafeteria. This implies that either staff are overestimating their ability to consume all the food taken or the time they have to eat, or that the food quality is not up to their expectation. This problem could be tackled with behavioural changes and/or changes to the menu.

This study aims at answering three main questions to tackle the root cause of food waste in the staff cafeteria: How much food should the chef be cooking each day? How to encourage staff to make better food choices and reduce edible food waste? How to provide feedback to the chef on potentially poorly cooked dishes?

The plan of the paper is as follows: “Method” describes the experimental methods used to address the three research questions stated above, namely upstream optimisation, midstream education and downstream recognition; “Results: a case study” describes the results of the experiments implemented in the integrated resort of this study; “Discussion and outlook” discusses the impacts of the interventions as well as challenges and improvements for future studies; finally “Conclusion” draws the conclusions of this study.

## Method

**Figure 2:**
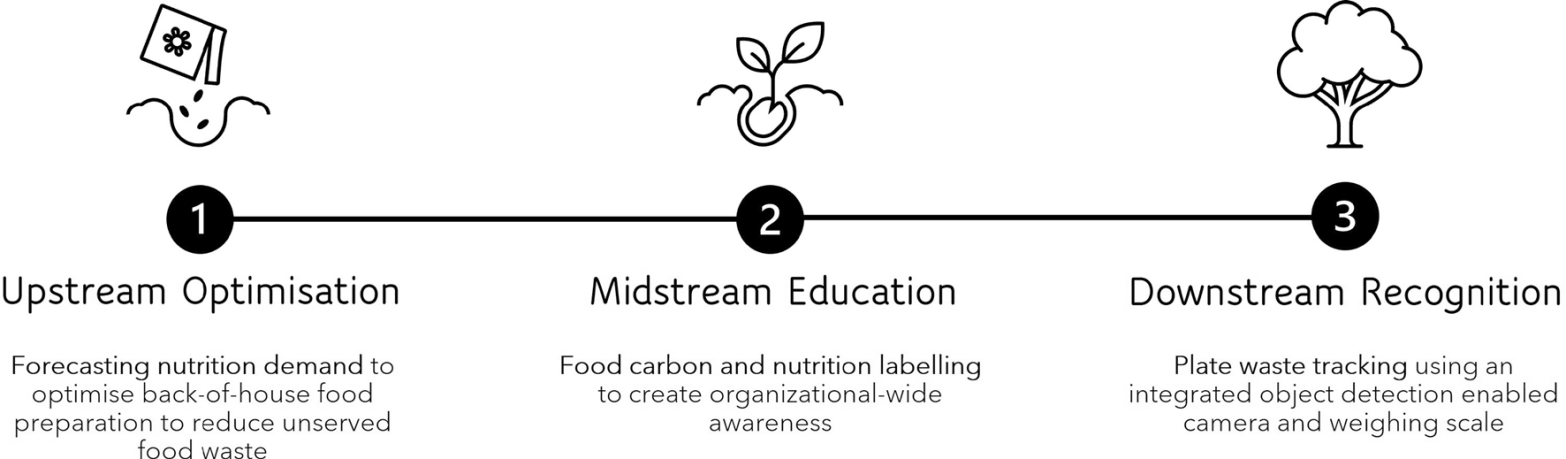
Three streams of decarbonisation for reducing life cycle GHG emissions from food waste: upstream optimisation, midstream education, downstream recognition.

Figure 2 shows the three streams of decarbonisation proposed in this study for reducing life cycle GHG from food waste. The three streams include: (1) upstream optimisation, (2) midstream education and (3) downstream recognition. The three streams make up a data-driven holistic approach to reducing food waste and carbon footprint of the integrated resort staff cafeteria. As the adage goes, ‘you can’t manage what you don’t measure’.

### Upstream optimisation: forecasting nutrition demand

Upstream optimisation aims to tackle the problem of unserved food waste. The source of the problem could stem from overcooking, poorly cooked dishes or poor quality ingredients leading to a lack of demand for the dish. To gain a better understanding of supply-demand in the staff cafeteria, the unserved food waste weight data from the back-of-house kitchen for different food categories were analysed and correlated with the number of covers served in the staff cafeteria on different days of the week to reveal a potential mismatch between supply and demand.

To model and forecast the demand in the staff cafeteria, hourly dining cover data were used. There were two years of historical data between 1st April 2020 and 1st April 2022 inclusive. The covers data showed strong daily and weekly seasonality due to regular meal times and weekends respectively. Thus, the Prophet package was well suited for this time series forecasting task^20^. The procedure was based on an additive model where a trend, seasonality, and holiday effects were fitted to the underlying non-linear data. It offered robust handling of missing data and shifts in the trend. The sources of uncertainty in the forecast included: uncertainty in the trend, seasonality estimates, and additional observation noise. The Prophet forecast model could be summarised by the following function

1 $$\begin{aligned} y(t) = g(t) + s(t) + h(t) + \epsilon (t), \end{aligned}$$

where *y*(*t*) is the output of the forecast, *g*(*t*) is the trend function which can be flat, a piecewise linear or a piecewise logistic trend, *s*(*t*) is the seasonal function which is approximated by a Fourier series to the intraday, weekly and/or yearly seasonal components, *h*(*t*) is the holidays function, and $\epsilon (t)$ is the error term which is assumed to be normally distributed. The seasonality mode could be additive or multiplicative. Multiplicative seasonal components are added as an amount proportional to the trend value *g*(*t*) at time *t*, such that seasonal effects respond proportionally to increases or decreases in the trend.

Having a model to forecast the number of covers in the staff cafeteria, the next step was to model how much food would be needed given the expected number of staff. This was achieved by modelling the human diet as composed of the following three macronutrients: Carbohydrates, fats, and proteins. The average recommended daily intake of 2000 kcal^21^ could be used to predict the amount of food needed. A study by Zhang et al.^22^ showed the difference in nutrient intake among Chinese subjects and other ethnic groups. They found that amongst the Chinese sample, the percentage macronutrient split between carbohydrate, fat and protein was roughly 50:35:15. Based on an online macronutrient calculator^23^, for a sedentary 35-year-old, 1.70 m tall, 60 kg male/female, who wants to maintain weight and intake moderate amounts of protein, the typical percentage macro split was 50:30:20. Given the common macronutrient calorie densities of 4 kcal/g for carbohydrate, 9 kcal/g for fat, and 4 kcal/g for protein, the equivalent grams per macro per day would be 250:67:100 for carbohydrate, fat and protein. This was treated as the baseline to satisfy the basic nutritional needs of an average staff.

Assuming the daily 2000 kcal was spread over three meals such that 20% was consumed at breakfast, 40% at lunch and 40% at dinner^24^ and that the percentage macro split remained the same as described previously, the amount of macros needed per meal would be

- Breakfast—50:13.4:20
- Lunch—100:26.8:40
- Dinner—100:26.8:40

where all ratios are given in units of grams for carbohydrate, fat and protein, respectively.

By forecasting the number of covers one day in advance, the chef could estimate a sufficient quantity of each dish to cook such that it would meet the basic nutritional needs of staff, minimising the potential for overcooking. The forecast model could be updated daily as new dining covers data come in at the end of the day.

### Midstream education: food carbon and nutrition labelling

Midstream education involves carbon and nutrition labelling all dishes in the staff cafeteria for staff to understand how their food choices impact the climate and their health. This required collecting the recipes for every dish served in the staff cafeteria, as well as the kitchen equipment used to cook the dishes to estimate the GHG emissions incurred in the consumption stage of the dish.

The menus in the staff cafeteria were organised into the following 13 types: beef, congee, egg and beans, dessert, fruit, pork, poultry, rice and noodle, sauce, seafood, soup, sweet soup, and vegetable. There were a total of 404 dishes and 344 unique ingredients from which the nutritional database was built.

For nutrition labelling dishes, nutritional data from Cronometer and sources therein were used. As most ingredient names were in Chinese, these were translated to English before building a custom nutritional database for the staff cafeteria dishes (see Supplementary Information). The amount of calories and macronutrients were reported in units kcal and grams per 100 g of the dish respectively, a convention in most nutritional labels.

For carbon labelling dishes, life cycle assessment (LCA) was used to estimate the total environmental impact of the food ingredients from production to disposal. By considering the energy and resources involved at each stage separately, it could help identify emission ‘hot spots’ such that targeted decarbonisation strategies could be implemented.

Five stages were included in the life cycle assessment of a food ingredient: (1) agricultural (for food production), (2) processing (including storage and packaging), (3) distribution, (4) consumption and (5) waste management. Each stage of the life cycle incurs a carbon footprint and should be accounted for when carbon labelling foods. In most cases, the agricultural stage makes the greatest contribution to the total food emissions, contributing 80–86% of food systems emissions, although with significant variations across regions and foods^25^. The remaining stages of supply chain emissions also vary drastically depending on food consumption patterns which differ by nation.

All raw ingredients were classified into one of 18 types: alcohol, aquatic, beef, dairy, egg, fruit, fungus, lamb, legume, maize, nut, oil, pork, poultry, rice, seasoning, vegetable, and wheat. This approach was adopted in order to use China-specific emission factors for certain food types based on a Beijing food waste carbon footprint study^26^. The sources of other emission factors, assumptions of energy intensity for food processing, transport modes and distances, and waste management emissions specific to Macau were documented in the emissions data files which are available in the Supplementary Information.

For each dish, the carbon footprint was given in kg CO2e/kg (i.e. kilograms of CO2-equivalent per kilogram of the dish) based on the weight of ingredients given in the recipe. CO$_2$ equivalent (CO2e) is a unit of measurement used to standardise the climate effects of various greenhouse gases (GHG) due to differing global-warming potential (GWP). The GWP is a measure of the warming effect over 100 years in comparison to CO$_2$. For example, methane (CH$_4$) is around 28 times stronger than CO$_2$, and nitrous oxide (N$_2$O) is around 273 times stronger than CO$_2$^27^.

To benchmark the carbon footprint of different dishes, the Swedish Meat Guide traffic light system was used, where $<4$ kgCO2e/kg, 4–14 kgCO2e/kg and $>14$ kgCO2e/kg were marked as green, orange and red respectively^28^. The threshold carbon footprint for food for an individual (or ‘fair daily food emissions value’) was set to 2.7 kgCO2e/day. This value was based on the 2030 total global CO2e emission needed to reach net zero by 2050 assuming a linearly decreasing trend, and can be calculated as follows: 33.8 gigatonnes CO2e/year/8.5 billion people/365 days $\times$ 25% (for food consumption) $\approx$ 2.7 kgCO2e/person/day^29^.

### Downstream recognition: plate waste tracking

Downstream recognition involves building a plate waste detection system using computer vision to better understand and quantify what staff are throwing away. It would allow the segregation of inedible (such as bones, shells, peels, etc.) and edible (such as vegetables, meat, rice, etc.) food waste. The detection of different food waste types could also inform the chef of potential changes to recipes such as cooking methods and/or adjust the ingredients accordingly.

One question that may arise is why plate waste cannot be sorted manually by the staff themselves. In an ideal world, this would be a viable approach to recognise and quantify plate waste accurately. However, reality presents several issues. For example, staff may be in a hurry and simply throw all their plate waste into a single bin. Plate waste could often be messy, so the segregation of edible and inedible food could be time-consuming and potentially lead to long queues in the cafeteria during peak times. It would require training thousands of employees to manually segregate their plate waste. Enforcing such an intervention may be a drain on human resources. On that note, an automated plate waste detection system would take human discipline out of the equation and help alleviate the problems described above.

The computer vision algorithm employed was a state-of-the-art object detection model called YOLOv5^30^, capable of detecting objects of interest in real time. Due to the large variety of food waste items, thousands of labelled food waste images were needed to provide a large enough dataset to train the model. To train an object detection model, two inputs were required: (1) images, and (2) labels consisting of bounding boxes with class names around all objects of interest in the images. As emphasised in the Machine Learning Engineering for Production (MLOps) course by DeepLearning.ai^31^, one should adopt a data-centric approach to machine learning. It is often the case that simple models trained with consistent and accurate labels beat the most advanced algorithms with inconsistent and inaccurate labels.

To annotate data, the Modified OpenLabelling tool^32^ and Roboflow platform^33^ were used to streamline the labelling process and collaborate with other annotators. The Modified OpenLabelling tool allowed for rapid labelling of images stored on the local machine. The Roboflow platform allowed for fine-tuning of bounding boxes and getting summary statistics of the dataset such as class distribution, which informed which classes were over- or under-represented so that data collection could be targeted towards under-represented classes to alleviate the problem of class imbalance.

Several metrics were used to quantify how well an object detection model performs. A common metric called intersection over union (or IoU) measures the overlap between two bounding boxes. It is defined as:

2 $$\begin{aligned} IoU = \frac{\text {Intersection Area}}{\text {Union Area}}, \end{aligned}$$

where ‘Intersection Area’ is the overlap between two bounding boxes, and ‘Union Area’ is the total area enclosed by both bounding boxes. A higher IoU means a larger fraction of overlap between two boxes, where a value of 1 means perfect overlap. If the IoU exceeds a predefined threshold (e.g. IoU = 0.5), then the overlap between a predicted and ground-truth bounding box is said to be a positive detection or a true positive (TP), otherwise, it is a false positive (FP). A false negative (FN) is if the model fails to predict a bounding box at all in an image containing objects of interest.

During inference, the model is given an unseen image and predicts bounding boxes. Each predicted bounding box has a probability (or confidence score) with a value between 0 and 1. A higher confidence value implies that the model is more confident that an object of a specific class exists at the predicted location. There may be many bounding boxes predicted initially by the model. To only keep the best bounding box around an object, YOLOv5 uses non-maximal suppression (NMS) to reduce the number of bounding boxes. Firstly, NMS will only keep the bounding boxes which have a confidence (conf) score greater than a predefined threshold (e.g. conf = 0.5) and remove them otherwise. Secondly, all predicted bounding boxes that have an IoU value greater than a predefined threshold (e.g IoU = 0.5) with respect to the best bounding box will be removed. A greater IoU between two predictions likely indicates two bounding boxes referring to the same object. This procedure is repeated for every predicted class in an image. The confidence and IoU thresholds used here are called NMS thresholds and can be set by the user before inference.

To measure how well the model predicts a given class, the precision and recall metrics are used to evaluate the predictions against the ground truth. Precision, which measures how accurate are the predictions, is given by

3 $$\begin{aligned} \text {Precision} = \frac{TP}{TP + FP}, \end{aligned}$$

i.e. the fraction of positive predictions (TP + FP) that is actually true (TP). Recall, which measures how well the model finds all the positives, is given by

4 $$\begin{aligned} \text {Recall} = \frac{TP}{TP + FN}, \end{aligned}$$

i.e. the fraction of positive instances (TP + FN) that is correctly predicted (TP). Here, *TP* is true positive, *FP* is false positive and *TN* is true negative. As the IoU threshold decreases from high to low, the precision would likely decrease and recall increase, because a lower IoU threshold is more easily satisfied thus more positive detections (*TP* or *FP*). Each IoU threshold would produce a different precision-recall (PR) curve for the labelled dataset. The average precision (AP) of each class is the area under the PR curve based on a single IoU threshold. It can be calculated as

5 $$\begin{aligned} AP = \sum _{k=1}^{k=n} (\text {Recall}[k] - \text {Recall}[k-1]) \times \text {Precision}[k], \end{aligned}$$

where *n* is the number of points in the PR curve and $\text {Recall}[0] = 0$. The points which make up a PR curve are model predictions ranked from high to low confidence down to a confidence threshold (e.g. conf = 0.25), under a single IoU threshold (e.g. IoU = 0.5). Interpolation is often used to have evenly spaced points. In other words, the AP of a class is a weighted sum of precision where the weight is the increase in recall between consecutive points. After calculating the AP for each class, the mean average precision (or mAP) can be calculated by averaging over all classes as

6 $$\begin{aligned} mAP = \frac{1}{n}\sum _{k=1}^{k=n} AP_k \end{aligned}$$

where $AP_k$ is the average precision of class *k*, and *n* is the number of classes. Note that the confidence and IoU thresholds used for evaluating AP and mAP are different to the NMS thresholds during inference.

For object detection, the two common metrics used to quantify the model’s performance are: $\mathrm {mAP@0.5}$ which means mAP with IoU = 0.5 over all classes and $\mathrm {mAP@0.5:0.95}$ which means mAP averaged over 10 IoU thresholds from 0.5 to 0.95 with a step size of 0.05 over all classes.

As a proof of concept, the small YOLOv5s model was trained for 1000 epochs with 250 plate waste images from the staff cafeteria captured manually using a camera with labelled food categories such as vegetables, meats and bones. The training was performed on an Nvidia V100 GPU in UCL’s Astrophysics high-performance computing (HPC) cluster. The model overfitted substantially to the training set, but it was nonetheless promising as the model was able to learn the underlying mapping between food waste images and food objects within.

To capture large amounts of food waste images efficiently whilst not interfering with the normal operation of staff, the following image collection mechanism was designed in the stewarding room. A steel mounting bracket was installed above the soiled tray return conveyor system, over 2 m high to prevent obstructing workers’ stewarding operation. A small clip-on camera was attached to the bracket looking directly below where workers would remove the soiled trays from the multi-tiered conveyor racks for waste disposal. The camera was set to video mode to continuously record for several hours. During post-analysis, frames were extracted from the videos at a rate of one frame per second. The frames were resized to 1280 $\times$ 1280 pixels to reduce the storage size. Using an active learning approach, the images were automatically labelled with an existing version of the model. The labels were manually checked and corrected as necessary before adding them to the existing training dataset on Roboflow for fine-tuning the bounding boxes, and finally retraining the model from scratch.

To determine the weight of the plate waste, a set of load cells was programmed to take two weight measurements before and after throwing the plate waste (just before the tray was removed). The weight of the plate waste could be found from the difference between the two weights, excluding the weight of the tray, plates, bowls, cups and utensils.

**Figure 3:**
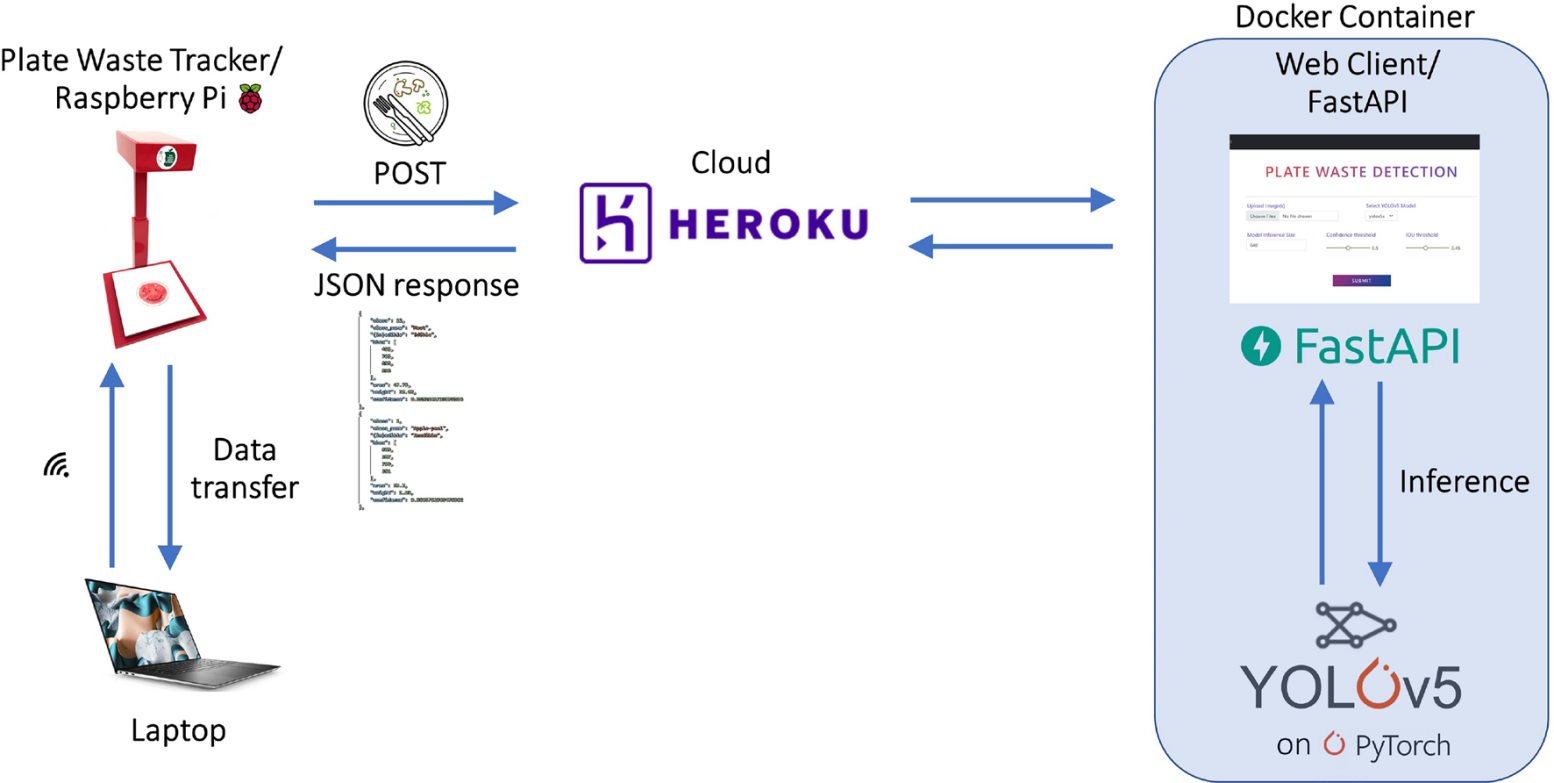
The plate waste tracker pipeline.

Building on the above ideas, a plate waste tracker prototype was constructed to incorporate all of the above. It consisted of: A Raspberry Pi (RPi) 3b+ unit for programming, two load cells for weighing, a camera module for capturing images, a wooden mounting stand to secure the two load cells with an acrylic platform on top, an overhang casing to house the RPi unit and the camera which was positioned directly above the weighing platform below. The two load cells were connected to the RPi general purpose input/output (GPIO) pins via a load sensors combinator. The camera module was plugged directly into the camera module port on the RPi board. The RPi.GPIO, hx711 and picamera Python libraries were utilized in a script to control the Raspberry Pi GPIO channels, and interface with the load cells and camera module. Upon switching on the plate waste tracker, the Raspberry Pi would establish a WiFi connection automatically and could be controlled remotely from a laptop via SSH (a network communication protocol). This allowed software updates to be pushed to the Raspberry Pi over-the-air. The final pipeline for the food waste tracker is illustrated in Fig. 3 and is summarised below:

1. A user puts a tray onto the tracker platform which triggers the ‘before’ weight and image to be captured. Both are stored in the Raspberry Pi’s SD card.
2. The image is uploaded via an HTTP POST request to the Heroku server which hosts the Docker container plate waste detection app^34^. The image will be processed by a cached YOLOv5 model for plate waste detection and the results will be automatically downloaded back onto the Pi SD card which could be displayed on a screen. Note that this inference step runs asynchronously (i.e. in parallel) with the rest of the program.
3. At the same time, the user disposes of their plate waste into a bin.
4. After disposal, the ‘after’ weight of the tray is captured automatically as the user removes the tray from the platform, at which point the weight of plate waste is calculated from the difference between ‘before’ and ‘after’.
5. This user’s plate waste data are stored in a CSV file for post-analysis.
6. The cycle repeats from step 1.

## Results: a case study

This study proposed three pathways to reduce food waste and the carbon footprint in the staff cafeteria of an integrated resort in Macau, addressing the questions raised in “Food waste in HaFS sectors and its associated impacts”. To determine how much food should be cooked, a staff cafeteria demand forecast and nutritional model were used to estimate the basic nutritional needs of staff in a day. To encourage staff to make better food choices, carbon and nutrition labelling were implemented for 404 dishes served in the staff cafeteria to raise awareness about the impacts of different foods on the climate and their health. To reduce edible food waste, a plate waste tracker equipped with computer vision was built to track what staff are throwing away, the statistics from which could inform the chef on potential poorly cooked dishes. The staff members who used the canteen on a daily basis were primarily in the Chinese ethnic group, between 30 and 50 years of age, possessing a high school or university education, a fairly even mix of males and females, with an average income of around $\pounds$ 25,000 per year (based on a MOP/GBP exchange rate of $\pounds$ 0.11 at the time of writing). The detailed results of each strategy are discussed below.

### Forecasting nutrition demand

**Figure 4:**
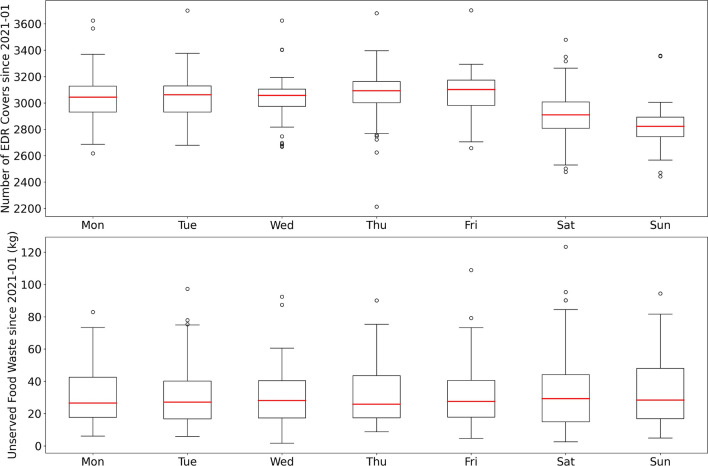
A box plot showing the staff cafeteria number of covers (top) and the weight of unserved food waste (bottom) for different days of the week. The bottom of the box is the lower quartile Q1 (25% of data below this value), the top of the box is the upper quartile Q3 (75% of data below this value), with a line at the median Q2 (50% of data below this value). The whiskers below and above the box extend no more than 1.5 $\times$ IQR (IQR = Q3–Q1) from the edges of the box, ending at the farthest data point within that interval. Outliers are plotted as dots.

Figure 4 shows the variation in the number of covers and the weight of unserved food waste across different days of the week. The results show a clear bimodal behaviour in the number of covers with weekdays high (typically $\sim$ 3100 covers) and weekends low (typically $\sim$ 2850 covers), due to office staff having weekends off. Generally, the average unserved food waste per day was $\sim$ 30kg across Monday to Sunday. Despite the average number of covers on Saturdays and Sundays being around 8% lower than on weekdays, the unserved food waste remained the same, if not higher on occasions as seen from the larger interquartile range (IQR). This result shows the potential to optimise the amount of food cooked based on dining covers forecast and macronutrient model to reduce unserved food waste.

**Figure 5:**
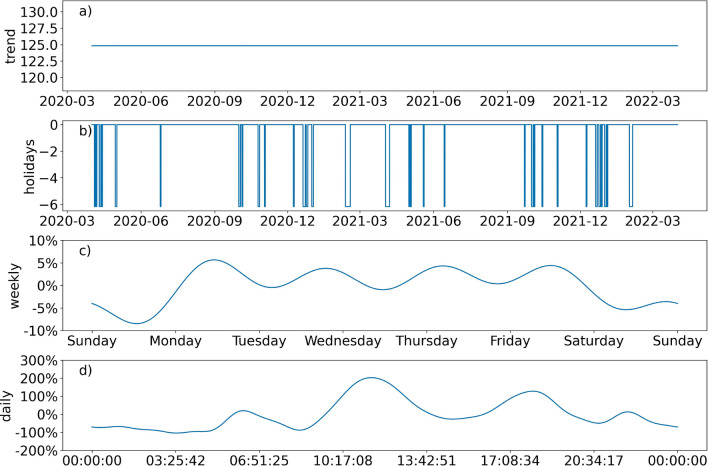
Components of the Prophet model used to fit the dining covers in the staff cafeteria over time. The panels from the top down are: (**a**) the trend (which is flat in this model) gives the base value of the forecast (in units of covers per hour), (**b**) public holiday effects which act to reduce the base value of the forecast, (**c**) and (**d**) weekly and daily seasonalities giving the percentage of the base value needed to add to the final estimate of the forecast.

Figure 5 shows the components of the forecast model for the number of covers in the staff cafeteria on an hourly basis. The best set of parameters was found to be: A flat trend (i.e. no growth or decline in the number of employees), including public holidays in Macau, a multiplicative weekly trend of Fourier order 5, and a multiplicative daily trend of Fourier order 15. These settings were found to produce the best forecasting performance on the test data with a mean absolute error of 18 counts, meaning on average in a given hour the difference between the predicted and true counts for the number of dining covers was 18 people of about 125 average hourly covers (see Fig. 5a).

**Figure 6:**
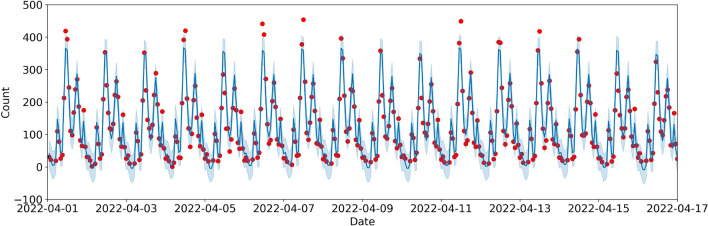
Forecast of the number of covers on an hourly basis for the period between April 01 and April 17, 2022 (inclusive). The blue line represents the forecast values, the light blue shade represents the 90% uncertainty interval for the counts, and the red dots represent the actual number of dining covers in a given hour.

Figure 6 shows the model applied to unseen test data over the month of April 2022 (only half-month shown for clarity). The forecast (blue line) with a 90% uncertainty interval in light blue captured well the weekly and daily patterns of the staff cafeteria covers in this test period. Other variations of the model such as without public holidays, or using linear piecewise trends, had a higher mean absolute error, despite visually producing better fits to the highest peaks during lunch hours.

**Figure 7:**
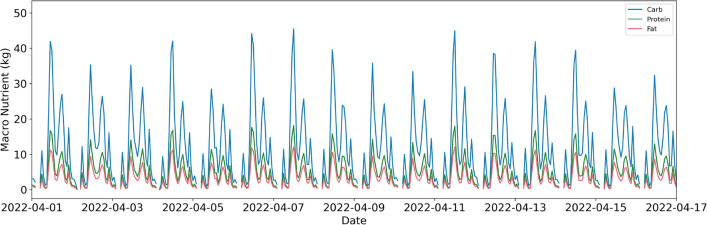
The macronutrient forecast on an hourly basis for the period between April 01 and April 17, 2022 (inclusive) based on the number of dining covers recorded. Each point represents the typical amount (in kg) of a particular macronutrient needed to meet the basic nutritional needs of staff members for that given hour.

Given a relatively accurate forecast for the number of covers, the nutritional needs of staff at a given time were also modelled. Figure 7 shows the nutritional model based on the number of staff at a given hour. It gives the recommended amount of macros needed to meet the average nutritional needs of staff as described in “Upstream optimisation: forecasting nutrition demand”. The daily amount of macronutrients required, based on a typical 2000 kcal diet per person, for some 3000 staff members on different days of the week was predicted to range between:

1. Carbohydrate: 245-280 kg
2. Fat: 71–75 kg
3. Protein: 98–112 kg

**Figure 8:**
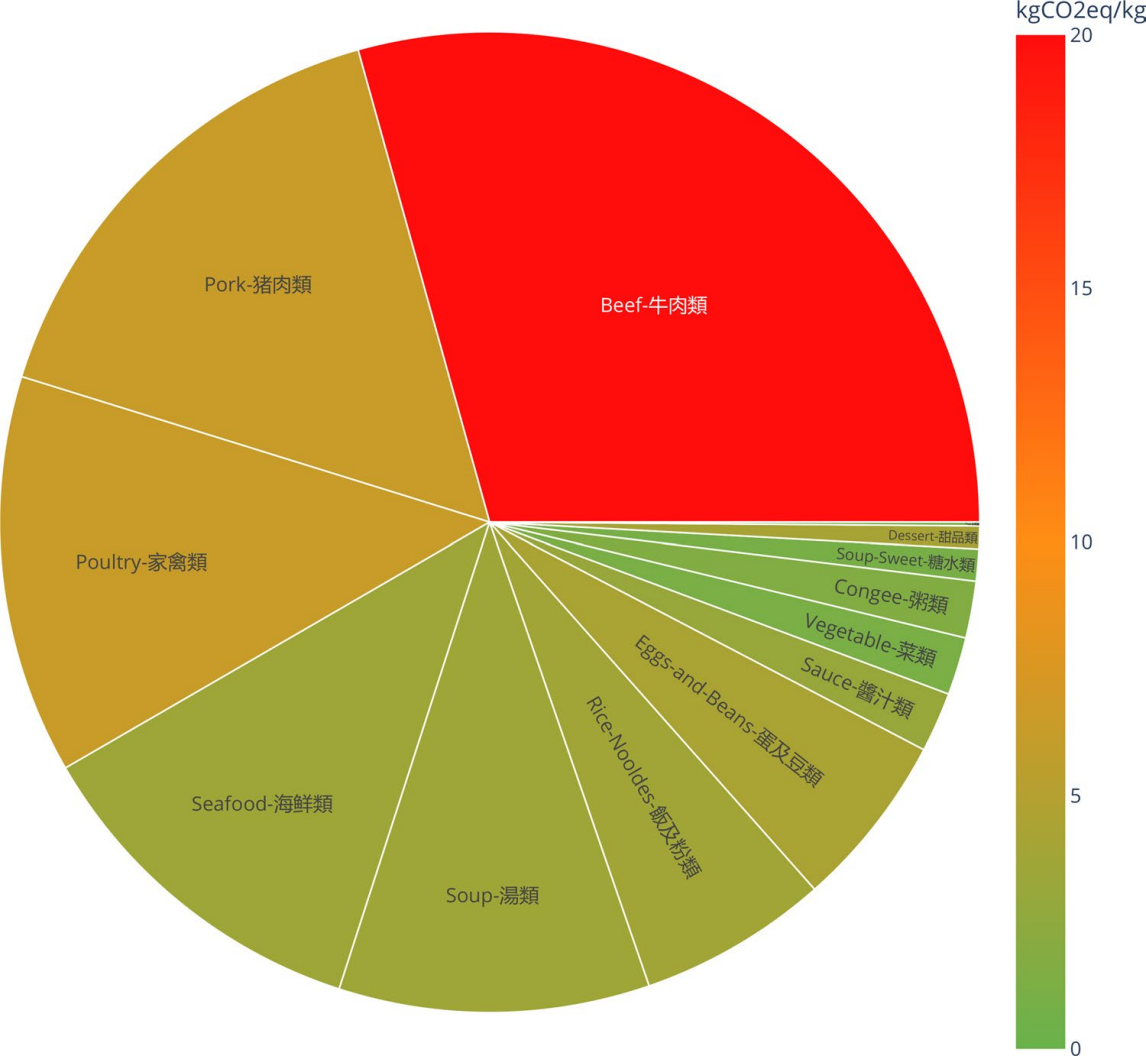
A sunburst plot showing the carbon footprint (kg CO2eq/kg) of 404 dishes grouped by their dish type. There are 13 dish types with the number of dishes indicated inside brackets: beef (28), pork (50), poultry (43), seafood (62), soup (62), rice & noodles (34), eggs & beans (26), sauce (14), vegetables (31), congee (25), sweet soup (22), dessert (4) and fruit (3). The fruit text label is not visible due to a very small sector size. The colour of each sector reflects the average carbon footprint in kgCO2e per kg of a dish in that sector.

Comparing these predicted macronutrient values with the actual amount served on a typical day, it was found that there were signs of oversupply. The amount of carbohydrates, fat, and protein supplied were 123%, 207% and 233% of the average predicted values respectively. Note that only the main dishes were included in this macronutrient analysis, dishes such as soups, sauces, and fresh salads were excluded from the calculation due to negligible amounts of macronutrients. However, both the nutritional and carbon footprint values were computed for these types of dishes as seen in Fig. 8. Their exclusion was purely on the basis of obtaining a baseline for the minimum macro-nutrients required. These results implied that the median 30 kg of unserved food waste per day could potentially be reduced significantly as there were more than 2 times the average required fat and protein on a typical day.

### Food carbon and nutrition labelling

**Figure 9:**
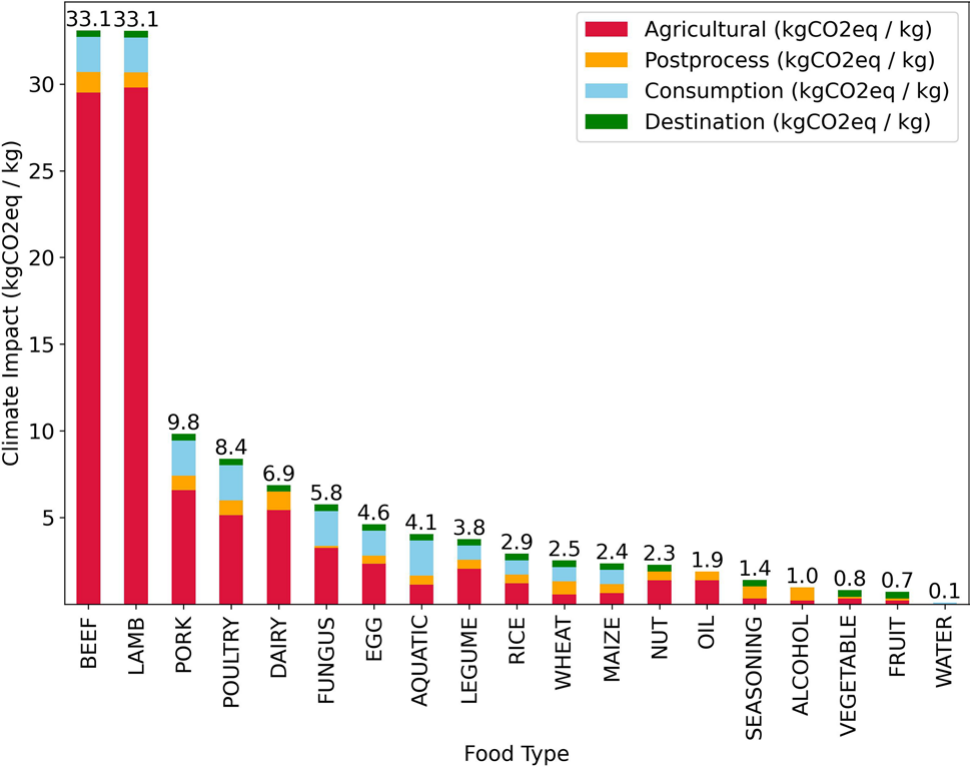
Full life cycle assessment of greenhouse gas emissions for different food types: agricultural, post-process (including post-harvest handling and storage and processing and packaging), consumption, and destination (i.e. waste disposal).

Figure 8 shows a sunburst plot of the carbon footprint of 404 dishes grouped by their dish type. When viewed in the browser^35^, the chart is interactive allowing different dish types to be expanded to view each dish with nutritional values . The size of the sector is proportional to the sum of carbon footprint in that sector. The colour of each sector is determined by the average carbon footprint in that sector and the scheme follows the Swedish Meat Guide traffic light system as described in “Midstream education: food carbon and nutrition labelling”. It is immediately obvious from the bright red section that beef dishes have the highest carbon footprint largely due to their high GHG emission in the agricultural stage of the life cycle as seen in Fig. 9.

The carbon label for each dish could be further correlated with different macronutrient values to link environmental (GHG) and personal health (nutrition) goals. Similar studies have been done in school canteens to connect the environmental impact of meals with their nutrition (e.g. Volanti et al.^36^). The goal of midstream education was to help raise awareness on how food choices and food consumption behaviour can lead to significant climate and health impacts^37,38^.

Based on the carbon and nutrition labelling work, a spin-off carbon and nutrition tracking website (called **ourfood**^39^ was custom-built specifically for the food served at the staff cafeteria. The purpose of this website was to give the staff members the ability to track their own carbon footprint and nutrition over time based on their food choices. It would also facilitate staff interaction with each other by comparing trends amongst themselves, fostering a healthy discussion about food, climate and nutrition. The user could set their own carbon and nutrition budgets/targets according to their needs with default values set to the recommended values based on the literature. The number of trees needed to offset the staff’s yearly food carbon footprint is also displayed based on the average annual CO2 offsetting rate of a tree^40^. Armed with this information, the company could direct a portion of their donations to fund tree-planting initiatives to offset the life cycle carbon emissions from the food served throughout the year, thus reducing the staff cafeteria’s carbon footprint.

### Plate waste tracking

**Figure 10:**
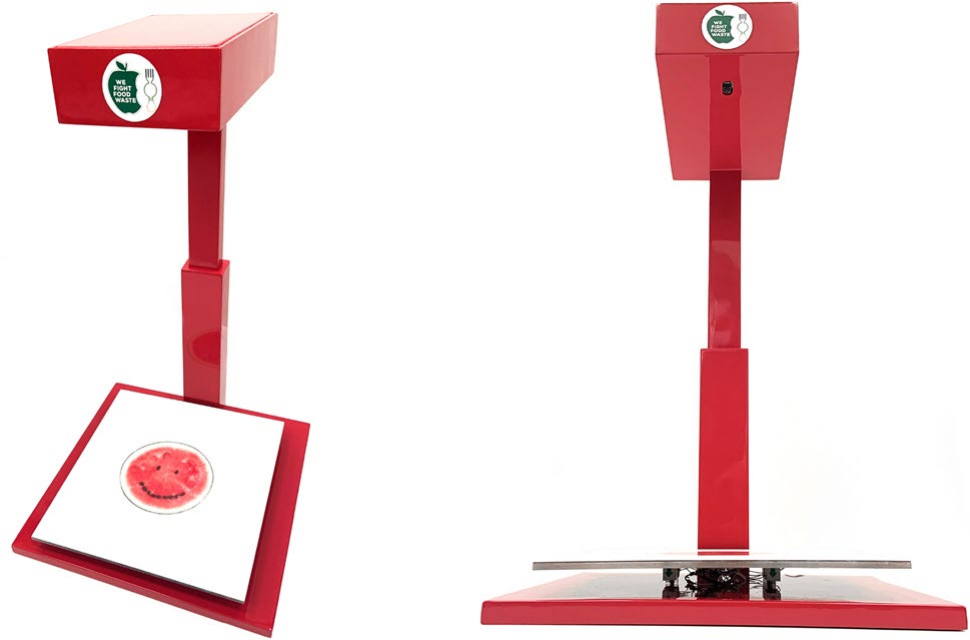
A prototype of an integrated plate waste tracker. It consists of load cells at the bottom to measure the weight of plate waste, and a camera at the top to capture an image of the plate waste before disposal, both of which are controlled by a ‘Raspberry Pi’ running a Python program.

Figure 10 shows the plate waste tracker prototype developed in-house to automatically capture individual plate waste images and weights. It was designed to seamlessly integrate with the planned staff cafeteria renovation in which staff would be required to dispose of their own plate waste (during which data is collected by the tracker) before putting it on the soiled tray return conveyor system.

For the computer vision component of the plate waste tracker, Table 1 shows the final performance of the small (s), medium (m) and large (l) YOLOv5 models to detect plate waste on the validation dataset across 32 classes including: apple, apple core, apple peel, bone, bone fish, bread, bun, egg hard, egg scramble, eggshell, egg steam, egg yolk, fish, meat, mussel, mussel shell, noodle, orange, orange peel, other waste, pancake, pasta, pear, pear core, pear peel, potato, rice, shrimp, shrimp shell, tofu, tomato and vegetable. The large model performed best unsurprisingly, with mAP@0.5 of 0.681 and mAP@0.5:0.95 of 0.439. However, the large model inference speed was around 3 times slower than the small model. The entire dataset had a total of 2716 images (10% of which was used for validation), with 10,531 object instances (i.e. labelled bounding boxes). As a comparison, the original YOLOv5 models were trained on the Common Objects in Context (COCO) dataset^41^ which contained 80 classes with 330,000 images and over 1.5 million object instances. The large model for this COCO dataset achieved mAP@0.5 of 0.711 and mAP@0.5:0.95 of 0.534.

**Table 1:** The performance of the small, medium and large YOLOv5 model on the plate waste dataset.

Model	Image size (px)	Epochs	nmAP@0.5	mAP@0.5:0.95
YOLOv5s	640 $\times$ 640	150	0.655	0.425
YOLOv5m	640 $\times$ 640	115	0.663	0.430
YOLOv5l	640 $\times$ 640	97	**0.681**	**0.439**

A likely reason for the poorer performance compared to COCO could be due to 15 under-represented classes with less than 100 instances. These classes contributed the highest amount of ‘background FN’ (i.e. lower recall, see Eq. (4)). On the other hand, over-represented classes such as bone, vegetable, eggshell, orange, orange peel, and apple peel contributed the highest amount of ‘background FP’ (i.e. lower precision, see Eq. (3)).

**Figure 11:**
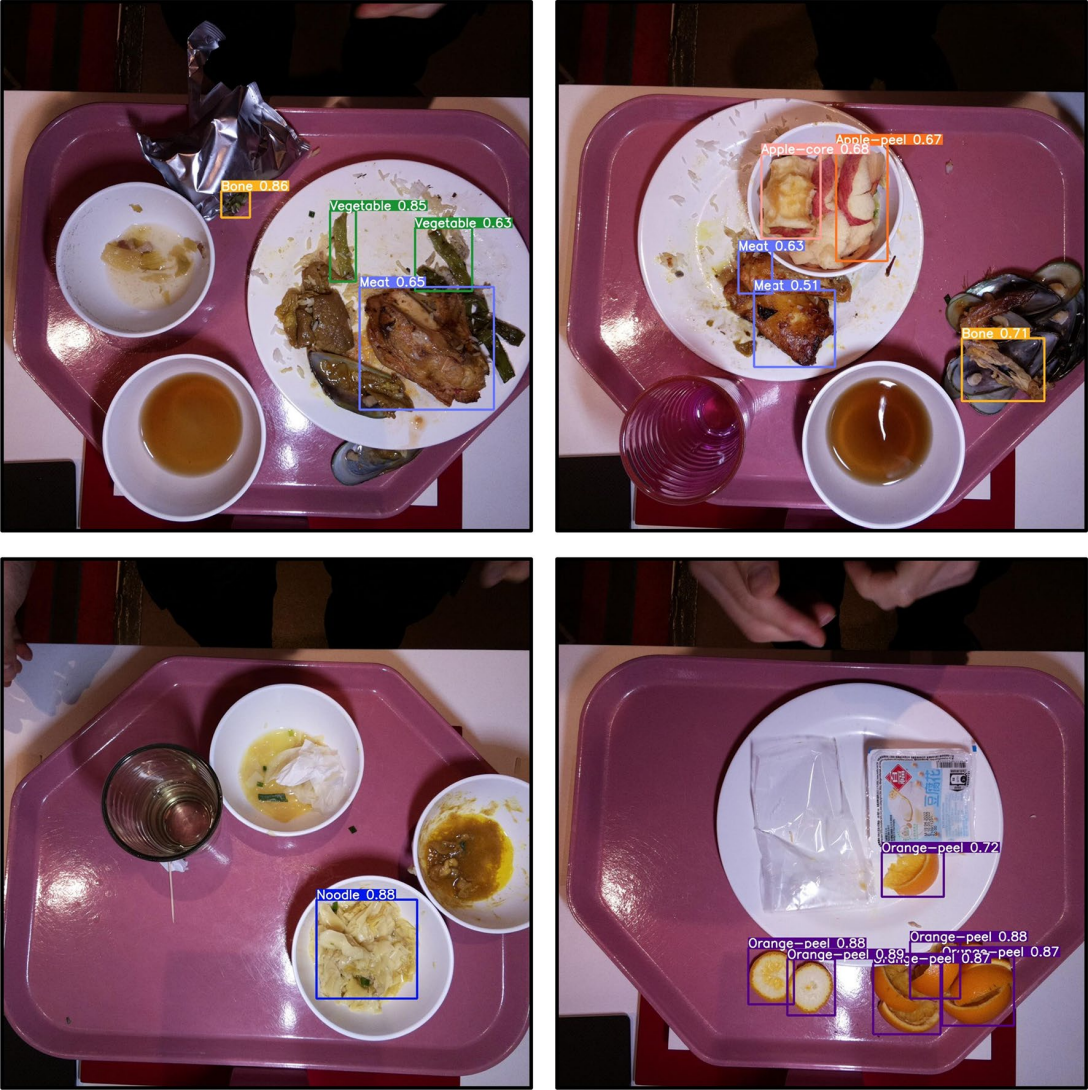
Example plate waste detection by YOLOv5m model on unseen test plate waste images captured by the plate waste tracker in Fig. 10.

Figure 11 shows an example of the YOLOv5m model predictions on unseen test examples of plate waste images collected by the tracker. It can be seen that for bulky food items, the model does a very good job of capturing the correct class and bounding box position. The integrated cam-weight system algorithm is able to measure the waste weight of individual trays independent of the different types of plates, cutlery, cups and bowls used. The core idea is that the system obtains an initial weight of the tray with both containers and waste. After emptying, the final weight of the tray and empty containers is obtained. Thus, the weight of the waste can be inferred from the difference in the initial and final weight values.

**Figure 12:**
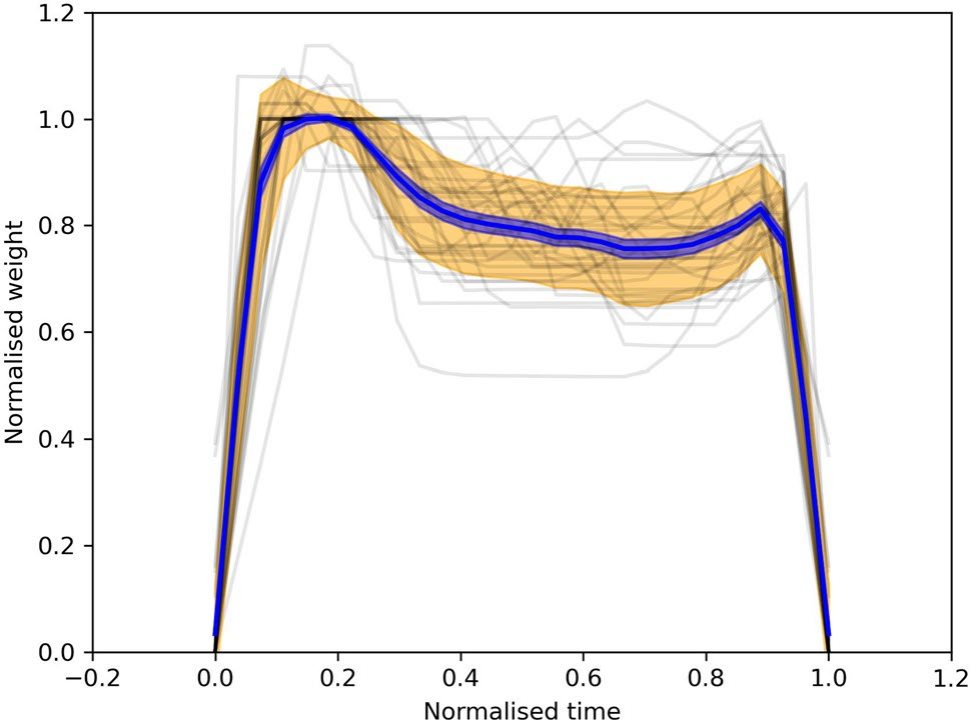
Superposed epoch analysis of 36 weight curves from plate waste disposal. The x-axis represents the normalised time where 0 is the initial placement of the food tray onto the weighing scale and 1 is the removal of the tray. The y-axis represents normalised weight where 1 is the 90th quantile value of the whole weight curve in a given sample.

A major benefit of individualised tracking of plate waste weight instead of a cumulative total is that it facilitates the calculation of different descriptive statistics which are far more insightful than a single total waste value. Figure 12 shows the epoch analysis of the weight curves from 36 samples of plate waste disposal in the staff cafeteria. The general spike feature at the end of the weight curve represents the placing of the bowl or plate back onto the tray before the removal of the entire food tray from the platform. Table 2 shows the summary statistics of the plate waste samples. On average, about 20% of the initial food tray weight consisted of plate waste, although there was a large variation between individual samples, ranging from 50 to 552 g, with a mean weight of 253 g per dining cover. For reference, the IFWC reported an average food waste of 115 g per dining cover^6^. A likely reason for the large discrepancy could be due to vastly different plate waste types between Asian and European cuisines. Since the plate waste remains unsegregated, the weight includes the weight of bones, peels and other inedible food parts. The computer vision component of the plate waste tracker attempted to partially solve this issue by detecting whether edible food was thrown away.

**Table 2:** Summary statistics of 36 samples of plate waste weight.

Statistic	Plate waste weight
Count	36
Mean (g)	253
Median (g)	255
SD (g)	131
95% CI of mean (g)	209–298
Min (g)	50
Max (g)	552
Range (g)	502

### Summary

A summary of the main results obtained for each of the three streams of decarbonisation and three interventions investigated in the case study is shown in Table 3.

**Table 3:** Summary of the main results for each of the three interventions investigated in the case study.

Intervention	Forecasting nutrition demand	Food carbon and nutrition labelling	Plate waste tracking
**Decarbonisation** **stream**	Upstream optimisation	Midstream education	Downstream recognition
**Main results**	The nutrition forecast model showed that there were more than two times the average required fat and protein on a typical day	An interactive carbon and nutrition tracking app was developed to help staff make less carbon-intensive and more nutritious food choices	An integrated plate waste tracker with a camera and weighing scale was built to automatically detect different types of food waste and quantify individual plate waste weight

## Discussion and outlook

In the upstream optimisation phase, this study showed the potential for chefs to optimise the food preparation based on the forecasted number of dining covers combined with a macro nutritional model, that is to prepare and cook only what and when is needed (just-in-time cooking method). Despite this, it is sometimes inevitable to over-prepare for logistic reasons and errors in the forecasting model. If unserved food cannot be used to serve people within the company (e.g. due to logistics and resource challenges), there are alternative options such as donating to external nonprofit organisations, sending it to animal feed, to anaerobic composting producing compost for new plants, to anaerobic digestion producing biogas for energy, and only finally to the bin for landfill or incinerator.

Food donation should be the first step to consider given that unserved food is perfectly good to eat for other people. However, chefs are often extremely busy preparing meals for thousands. The barriers to entry should be minimised to encourage participation in the joint effort to reduce food waste. There needs to be infrastructure to make donating an easier option than disposing of unserved food directly to the bin. For example, the Chefs to End Hunger^42^ non-profit foundation simplifies surplus food recovery by picking up boxes of surplus food from customers daily upon regularly scheduled product delivery for redistribution to different non-profit organisations.

In the midstream education phase of this research, an interactive carbon and nutrition tracking app has been developed to help diners better fill their tray by better informing them about the health and environmental impacts of food.

For nutrition labelling, each macronutrient was treated as a separate quantity. However, different macro- and micro-nutrients could be combined into a single nutrient density index for the dishes which captures various factors of nutrition^38^. However, this required the micro-nutrient quantities for each ingredient, which was beyond the scope of this study.

For carbon labelling, the carbon footprint of a dish was only one indicator of the environmental impact of food. The water footprint (i.e. the amount of water used) and ecological footprint (i.e. amount of biologically productive area used) are also important environmental indicators which measure the impact of food on the planet. In developed countries, diets rich in processed foods, meats and dairy products could have a greater climate impact than developing nations which consume a more local plant-based diet. A recent study showed that the environmental impacts of the Brazilian diet are increasing (based on the three environmental indicators) while the amount of ultra-processed foods has also increased^43^. It is necessary to shift consumption patterns away from diets rich in GHG-intensive foods without compromising nutritional values^44^. This reiterates the fact that dietary choices have serious environmental implications. It is through education such as carbon and nutrition labelling to inform people to make better food choices for their health and the environment.

Regarding the plate waste tracker, some improvements could be implemented for a more user-friendly experience. For example, a white LED may indicate when the plate waste image is captured before the user starts to dispose of the waste. Some visual guides and signs may be helpful too. A limitation of the current logging algorithm is that it prevents users from removing the tray from the platform to dispose of waste, due to the weight of the tray acting as a trigger for the algorithm. Once the tray is removed, it is assumed that the user has finished disposing of their waste and the logging cycle ends for that user. A potential solution could be to introduce another piece of crockery, for example, an easy-to-wash silicon container that moulds to the sides of the tray and is specifically used for inedible solids for easy disposal later. This may also make the detection of edible food easier for the computer vision system as it would not be obscured by other inedible parts. For the computer vision capability, different versions of the models could be trained by removing the under-represented classes. This is expected to increase the mAP scores due to better recall scores across all classes with sufficient instances.

Furthermore, from a psychological perspective, the midstream education and downstream recognition aspect of this study could be framed as nudging. Different types of nudging elements could be created from these tools to provide positive reinforcement and indirect suggestions as ways to influence the behaviour and decision-making of staff. In psychology, a seminal book written by Thaler proposed that the human brain makes decisions in two modes: one, which is intuitive and automatic so it is fast and almost effortless, and another, which is reflective and rational so requires high concentration and effort to execute^45^. More recently, Kahneman dubbed these two modes as System 1 and System 2 thinking respectively^46^. System 1 (automatic) nudge affects behaviour directly whilst system 2 (reflective) nudge affects choice directly^47^.

**Figure 13:**
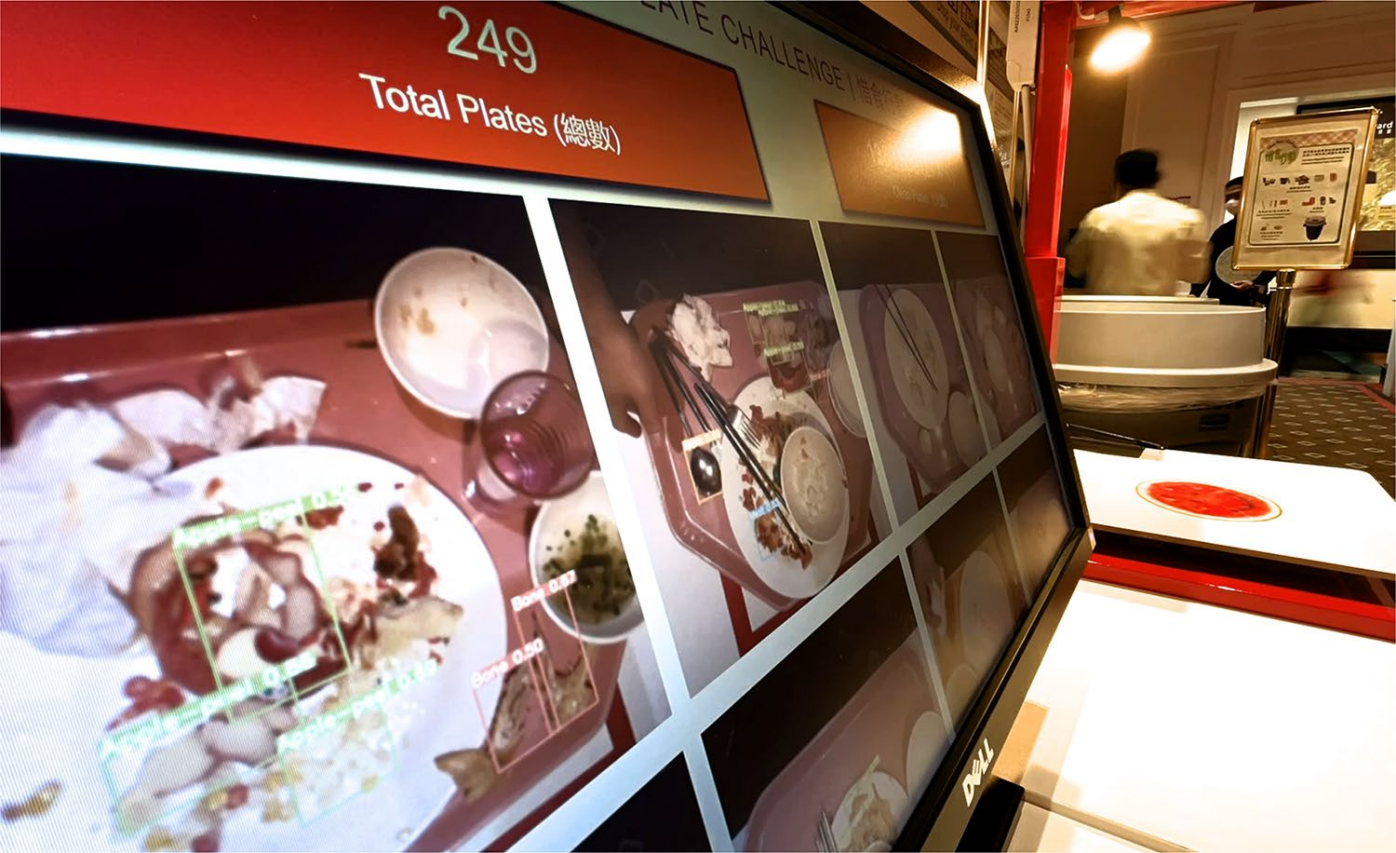
Clean plate challenge real-time display. It shows a live count of the total number of plates, the number of clean plates (with percentage to target) and the images of trays with bounding boxes drawn.

In the context of the carbon and nutrition labelling of foods, it provides the information needed for staff to make more informed food choices which benefit their health and the environment. Eventually, this may form part of the decision process when deciding what food to eat and could influence their purchasing and food consumption behaviour outside of the company too. In the context of the plate waste tracker, an array of LEDs and/or sounds could be triggered based on the results of the plate waste detection. For example, red LEDs may flash rapidly (with fast repeated beeps) when the number of edible food detected exceeds a certain threshold. Otherwise, green LEDs light steadily and have a simple sound effect. The flashing red LEDs and fast beeping sound would discourage wasteful behaviour as these stimuli are unpleasant (a negative punishment). On the other hand, the green LEDs and simple sound effects after a clean plate lead to positive reinforcement and encourage staff to maintain this behaviour. The plate waste tracker could be leveraged to introduce gamification by providing a live count of the number of clean plates in an automated manner coupled with image detections displayed in real-time on a monitor in the staff cafeteria. The objective, for example, could be to achieve a target number of clean plates by the end of the week to unlock a certain prize and for a weekly draw. The progressive nature of a score creates strong engagement for staff whilst giving them a sense of collaboration. Everyone has a role to play and contribute to the number of clean plates! This idea was implemented during a recent initiative by the company called the ‘clean plate challenge’ as shown in Fig. 13. It added entertaining and appealing elements which motivated staff to pay more attention to a usually neglected problem of food waste. Furthermore, this system provided another means of efficient food waste image data collection, as merely three hours of deployment yielded over 700 images. Gamification has been used in other settings such as enhancing physical activity participation^48^. Literature reviews have shown that gamification can increase user motivation and engagement but does greatly depend on the context of the issue, as well as on the users using it^49^.

A further study could evaluate the effectiveness of the three pathways to decarbonise the staff cafeteria in other integrated resorts in Macau. The combined workforce employed by the ‘Big Six’ gaming concessionaires is around 100,000 strong as of Q4 2021 (estimated from the annual and financial reports in 2021 of Galaxy, Melco, MGM, Sands China, SJM, and Wynn). Crucial statistics gathered over a period of time would enable a comparison of food waste quantity and associated carbon footprint with baselines before such interventions were in place. Furthermore, if these strategies proved to be effective, they could be implemented across educational institutions in Macau which has a combined total of over 133,000 students from preschool to higher education in the academic year 2020–2021^50^ of which around 31% are in higher education and 69% are in non-higher education, many of which provide catering services. The implementation of decarbonisation strategies at these large corporations and institutions would set the bar for smaller businesses in Macau. Other decarbonisation innovations may arise in the process which would be a step in the right direction to drive a new wave of environmentally responsible businesses.

Much of the data work is to inform the narrative. Seeing thousands of images of perfectly good food being thrown into the bin makes it very real. After all, this is a human problem and if data can help change people’s perspective on the enormity of the food waste problem, then everyone is inspired to act and achieve more ambitious goals. This can be viewed as nudges based on social norms (norm-nudges) and can be compelling behavioural interventions. Cutting-edge technology to improve the traceability of our food system is one approach to reducing food waste, but the basic human-focused strategies should not be forgotten. A holistic approach is one which tackles all aspects of this complex system, so no one part is sufficient on its own to solve the food waste problem.

Food waste has environmental, social and economic implications. Food that ends up in the waste stream requires resources to manage its diversion and disposal, not to mention the financial loss as edible food has monetary value too. Hence, reducing food waste directly translates to financial savings. Research and interventions needed to reduce food waste may be a financial burden to begin with. However, in the long run, a successful reduction in food waste would likely result in monetary gain for the organisation. More importantly, reducing food waste should be seen as a corporate social responsibility. As one of the six big gaming concessionaires in Macau, it is increasingly important that it plays its role to support China’s National Dual Decarbonisation Goals^51^ and to holistically integrate the Environmental, Social, and Governance (ESG) principles in the business operation due to expectations and pressure from internal and external stakeholders.

## Conclusion

This study implemented three decarbonisation pathways in the staff cafeteria of an integrated resort in Macau as a real-life case study for reducing life cycle GHG emissions from food waste:

1. Upstream optimisation was used to optimise the food supply based on a demand forecast model for the number of dining covers expected at a given time combined with a macronutrient model. This strategy is expected to help reduce unserved food waste by creating a responsive system in which the food supply matches the demand.
2. Midstream education was used to help raise awareness between food choices and their impact on the climate and health by providing carbon and macronutrient labels on all dishes served in the staff cafeteria. This intervention facilitated system 2 (reflective) nudge which aims to steer people towards a lower carbon and healthier food choices.
3. Downstream recognition was used to help reduce edible food waste by using a state-of-the-art computer vision model to detect plate waste in real-time and feedback to the user via a display to increase engagement. This intervention facilitated system 1 (automatic) nudge which aims to encourage users to reduce edible food waste.

## Supplementary Information

Supplementary Information 1.

Supplementary Information 2.

Supplementary Information 3.

Supplementary Information 4.

## Data Availability

All data were collected from the Wynn Macau property with help from various teams. The datasets created and analysed during the current study are available from the corresponding author upon reasonable request.
